# Identifying Systemic Lupus Erythematosus From Serum Proteomic Profiles Using Machine Learning and Genetic Risk Stratification

**DOI:** 10.1002/art.70156

**Published:** 2026-05-04

**Authors:** Mehmet Hocaoǧlu, Jishnu Das, Amr H. Sawalha

**Affiliations:** ^1^ Division of Rheumatology and Clinical Immunology, Department of Medicine University of Pittsburgh Pittsburgh Pennsylvania; ^2^ Lupus Center of Excellence University of Pittsburgh School of Medicine Pittsburgh Pennsylvania; ^3^ Center for Systems Immunology, Departments of Immunology and Computational & Systems Biology University of Pittsburgh Pittsburgh Pennsylvania; ^4^ Division of Rheumatology, Department of Pediatrics University of Pittsburgh Pittsburgh Pennsylvania; ^5^ Department of Immunology University of Pittsburgh Pittsburgh Pennsylvania

## Abstract

**Objective:**

Proteome‐wide risk models for lupus remain underexplored. We developed classification models to identify lupus from serum proteomic profiles.

**Methods:**

Patients with lupus and individuals with other autoimmune diseases in the UK Biobank were included. Differential proteomic expressions were characterized and followed by hierarchical clustering analysis. Proteomic linear and machine learning models were developed for established disease classification and future lupus prediction and compared to polygenic risk scores. Two additional independent lupus cohorts from Sweden, as part of the Human Protein Atlas (HPA), and from China were used for replication.

**Results:**

In the UK Biobank, 44,173 participants with proteomic data, including 2,063 individuals with at least one autoimmune disease at enrollment, were studied. This included 383 patients with lupus with a mean age of 43.6 ± SD 11.7 years at disease onset. Lupus showed the largest number of dysregulated proteins among autoimmune diseases and clustered with rheumatoid arthritis. Comparison with HPA showed that ~70% of lupus proteomic associations could be replicated with moderate correlation in effect sizes. The machine learning model outperformed the linear model in identifying pre‐existing lupus and generalized well to future lupus prediction. Among patients with lupus on immunomodulatory medications, the model reached ~90% sensitivity at 95% specificity, which was replicated in an independent cohort. Model interpretation highlighted SCARB2, SOD2, CD302, Galectin‐9, and GGT5 proteins with substantial effects on lupus identification.

**Conclusion:**

Proteomic machine learning models show excellent performance for identifying pre‐existing lupus and generalize well for predicting lupus before clinical diagnosis. Model interpretation identified novel candidate biomarkers for lupus.

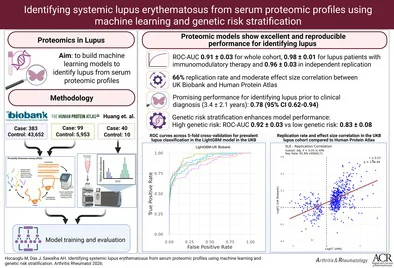

## INTRODUCTION

Systemic lupus erythematosus (SLE or lupus) is a complex autoimmune disease characterized by heterogenous clinical presentations with substantial disease burden in the population.^1^, ^2^ There are significant delays in lupus diagnosis, which can lead to poor outcomes.^3^, ^4^ This creates an important need to develop tools for future lupus risk prediction and improved diagnosis of established disease.^5^

Polygenic risk scores (PRSs) and genetic–environmental models have been developed to characterize lupus risk, but their performances are modest, limiting their utility.^6^, ^7^, ^8^, ^9^ Recently, proteomic risk scores based on a small number of candidate proteins were developed to estimate future lupus risk with limited accuracy.^10^, ^11^ However, comprehensive proteome‐wide models for identifying pre‐existing disease and improved risk prediction are lacking. In addition, existing risk prediction tools for lupus have largely been limited to linear approaches. Machine learning methods could potentially improve model performance by capturing the complex interactions between genes and proteins.^12^, ^13^ Further, machine learning model interpretation through feature importance analysis can help reveal candidate biomarkers with potential functional relevance.^13^, ^14^, ^15^

Previous research showed that lupus pathogenesis expands across multi‐omics layers with dysregulation observed in genomics, epigenomics, proteomics, metabolomics, and others.^16^ Hence, integrative approaches in risk modeling across different omics layers could lead to improved prediction models, but there is insufficient research in this area. Although proteomic dysregulation is well established in lupus, there is limited research on how lupus proteomic signatures compare to those of other autoimmune diseases. In this study, we aimed to characterize the proteomic signature of lupus relative to other autoimmune diseases and build classification models for lupus based on serum proteomic profiles using machine learning and genetic risk stratification.

## PATIENTS AND METHODS

### Study population and design

UK Biobank is a prospective cohort study that enrolled around 500,000 individuals aged 40 to 69 years between 2006 and 2010. Participants underwent whole genome sequencing and had their medical records in the United Kingdom linked with their research records. A subset of the UK Biobank cohort underwent proteomic profiling through the Proximity Extension Assay method of the Olink Explore platform. This platform provides normalized protein expression values representing relative quantification of serum proteins on a log2 scale. Details of the UK Biobank study design and Olink methodology have been published elsewhere.^17^

### Case selection and quality controls

We included all individuals who had a diagnosis of the following autoimmune diseases in the UK Biobank: SLE (M32), multiple sclerosis (G35), rheumatoid arthritis (M05 and M06), ankylosing spondylitis (M45), inflammatory bowel disease (K50 and K51), and other inflammatory spondyloarthropathies (M46). The following diseases were not studied despite being autoimmune in nature as there were fewer than 50 prevalent cases available after removing missing data: systemic sclerosis (M34), psoriatic and enteropathic arthritis (M07), dermatopolymyositis (M33), and polyarteritis nodosa and related conditions (M30). The demographics of the cases and controls were obtained from the UK Biobank enrollment survey. Self‐reported medication use was obtained from the verbal interview during the nurse visit at the cohort entry. Self‐reported ethnic background of the participants was defined as the following populations with their respective subpopulations by the UK Biobank study protocol (White: British, Irish, any other White background; Asian or Asian British: Indian, Pakistani, Bangladeshi, any other Asian background; Black or Black British: Caribbean, African, any other Black background). As the sample size of the remaining populations in the survey (“mixed,” “Chinese,” “other ethnic group,” “do not know,” “prefer not to answer”) was limited, they were grouped into a separate category as “other populations.” A prevalent case was defined as having its first diagnosis date before or within six months of the sample collection date for proteomic profiling. For controls, we used all the individuals with proteomic data after excluding the incident cases for the respective disease being analyzed.

For quality controls, data were preprocessed by the UK Biobank Pharma Proteomics consortium^18^ by removing the outlier samples, defined as being more than five SDs away in any of the following: principal component analysis and sample median and interquartile range. Samples with likely sex swaps indicative of manual handling errors and those with assay warnings by the manufacturer were also removed. As an additional quality control, we removed samples and proteins with more than 20% missing data from our analysis. Further details regarding case selection, data processing, and quality controls are described in Supplementary Material 1.

### Hierarchical clustering based on serum proteomics

We performed a case‐control analysis for each disease included in our study to compare the relative expression level of each protein. Only prevalent cases with respect to the sample collection date were included in this analysis whereas incident cases were excluded. We modeled the disease as a binary variable and included age, sex, population, batch, and time of sample processing as covariates in the model. Missing data for each protein were imputed with their respective medians across all samples for this analysis. Subsequently, linear models were fit with empirical Bayes moderation of SEs.^19^ Differential expression statistics were obtained in the form of log fold changes (logFCs) with their associated moderated t‐statistics and *P* values for each protein. False discovery rate (FDR) was controlled at 5% through the Benjamini‐Hochberg approach.^20^ To cluster proteomic signatures across diseases, we assembled a matrix of moderated t‐statistics values, with rows corresponding to proteins and columns to diseases. We computed pairwise distances of the diseases based on the Euclidean distances of the t‐statistics profiles and performed hierarchical clustering using the Ward method.^21^ These statistical analyses were conducted in R using the limma package (version 3.60.6), with default parameters.^19^

### Comparison with the Human Protein Atlas lupus cohort differential abundance analysis

We compared our results with the summary statistics from the differential abundance analysis in the Human Protein Atlas (HPA) lupus cohort, which underwent proteomic profiling through the Olink Explore platform. This analysis was performed by comparing the serum proteomic expression of 99 patients with lupus (enrolled in the Karolinska Institute in Sweden) to 5,975 controls. The details regarding the HPA data collection and analysis have been published elsewhere.^22^ We calculated the replication rate between HPA and UK Biobank by calculating the percentage of the proteins that showed a significant association with lupus in HPA with an FDR less than 5% and had replication in the UK Biobank. Replication was defined as having a nominal significance of a *P* value less than 0.05 and consistent effect size direction in both data sets. We also calculated the Pearson correlation of the effect sizes (logFC) across both cohorts to assess the generalizability of the proteomic signature.

### Hyperparameter optimization and performance evaluation of proteomic classification models

We developed separate classification models using proteomic expression data as input features and binary disease status as target label for each disease. A logistic regression model served as our linear baseline, with hyperparameter optimization performed across two regularization approaches for each disease separately: L1 (Lasso) and L2 (Ridge). Hyperparameter optimization is essential for maximizing the model performance by systematically identifying the parameter configurations that best balance model complexity with predictive accuracy on unseen data.^23^

For our machine learning approach, we used LightGBM, a gradient boosting framework that employs a leaf‐wise tree growth strategy.^14^ This model was selected based on the established state‐of‐the‐art performance of the gradient boosting methods on medium‐sized tabular data sets analogous to our own.^24^ LightGBM hyperparameter optimization was achieved through a two‐stage hyperparameter tuning process. We performed an initial random search across a broad range of parameters,^25^ followed by focused optimization using Bayesian search strategies.^26^ We employed weighted loss functions to address class imbalance by adjusting sample weights in inverse proportion to class frequencies. As the focus of our study was lupus, we trained the machine learning model for lupus classification. Further details regarding hyperparameter optimization processes are described in Supplementary Material 2.

Model performance was assessed through five‐fold stratified cross‐validation, with stratification based on disease prevalence, age, sex, and population (ethnic background) to ensure representative sampling across folds. This analysis was restricted to the prevalent cases. Median imputation was performed using only the training data within each fold. For a sensitivity analysis, missing data were not imputed leveraging the LightGBM's native missing data handling methods.^14^ Performance metrics for model evaluation included area under the receiver operating characteristic curve (ROC‐AUC), area under the precision‐recall curve (PR‐AUC), and max F1‐score. We also calculated sensitivity at a 5% false positive rate threshold. The mean and SD were calculated across all folds for all performance metrics and were written as mean ± SD format throughout the text. Final hyperparameter selection was based on the mean PR‐AUC performance across five folds. PR‐AUC was selected as the goal metric due to its superior sensitivity for the positive class performance compared to ROC‐AUC in highly imbalanced classification problems such as ours, in which the goal is to identify rare disease occurrence in the population.^27^ Delong's test was used to statistically compare the differences in model discrimination using out‐of‐fold predictions.^28^

For additional sensitivity analyses, we evaluated the model performance separately for patients with lupus with or without self‐reported immunomodulatory medication use and those with or without primary care record–based diagnoses (Supplementary Material 3); we applied leave‐one‐batch‐out cross‐validation, holding out each batch in turn for testing to assess potential batch effects; and we performed nested five‐fold cross‐validation to assess for potential optimism in model performance from hyperparameter selection.^29^, ^30^ These analyses were conducted in Python version 3.12.9 using scikit‐learn version 1.7.1 ^31^ and R version 4.4.2.

For independent replication, we tested the model performance using the individual level data from a lupus cohort reported by Huang et al.^16^ This cohort included 40 patients with lupus and 10 healthy controls who underwent proteomic profiling through liquid chromatography‐tandem mass spectrometry on an Orbitrap Fusion Lumos machine in data‐dependent acquisition mode. As only a subset of proteins measured in the UK Biobank were also measured in this data set, we retrained our model using only the overlapping proteins. We used the whole UK Biobank cohort for training and tested the model performance on the data set from Huang et al following the same strategy as described above. Due to cross‐platform measurement shifts and platform‐dependent missingness rates, we recalibrated the mass spectrometry–based measurements onto the Olink platform scale using a randomly selected calibration set from the mass spectrometry data from controls, which were excluded from model evaluation. Further details regarding the data alignment approach are described in Supplementary Material 4.

### Construction of a PRS and genetic risk stratified prediction for lupus

We used the joint sample whole genome sequencing data of the UK Biobank for constructing a PRS using the 176 previously identified genetic risk variants for lupus as we previously described (Supplementary Table 1).^6^ We restricted this subgroup analysis to the individuals who self‐reported as being White due to the differences in the average lupus PRS across different populations and smaller sample sizes precluding population‐specific analyses for other populations.^32^ Further, we removed 20 samples that failed quality controls during data generation by the UK Biobank.

We defined low, medium, and high genetic risk groups based on the tertiles of the lupus PRS. We then tested the performance of the LightGBM model on each genetic risk group separately using stratified five‐fold cross‐validation as described for the main analysis except that we also stratified by the PRS groups. We also evaluated the performance of the PRS‐only model using logistic regression for identifying all lupus cases in the UK Biobank, as genetic risk is fixed at birth.

Next, we searched for overlaps between lupus risk variants included in the PRS and previously identified protein quantitative trait loci (pQTLs) in the UK Biobank, including pQTLs that are within 100 kb of each PRS variant to capture the relevant linkage disequilibrium block.^33^ We used the pQTL analysis results reported for the combined cohort (all populations) and a *P* value threshold of 1.7 × 10^−11^, which is the genome‐wide significance threshold 5 × 10^−8^ adjusted for 2,923 unique proteins measured in the UK Biobank as defined by the authors of the original analysis.^33^

### Feature importance and ablation analyses

We performed feature importance analyses both on the LightGBM machine learning model and logistic regression to identify the most influential proteins affecting prediction performance. For logistic regression, we calculated the average coefficients across five folds for each protein to estimate feature importance. For LightGBM, we performed Shapley additive explanations (SHAP) analysis to identify the top 20 most influential features.^15^ We also obtained the top 20 features per the split and gain importance per LightGBM model metrics.^14^ Spearman correlation was used to compare the rankings obtained by each metric. These metrics provide information on which features influence the model the most when it makes a prediction.^14^, ^15^ However, as the model prediction itself could be wrong, we examined the empirical effect of these features (ranked within the top 20 by any of these three metrics) on model performance through a leave‐one‐feature‐out approach.^34^ This method is applied by sequentially removing each feature from the analysis and retraining the model and then calculating the actual change in PR‐AUC compared to full model performance.

### Proteomic model performance for lupus classification before clinical diagnosis

We tested the performance of the machine learning (LightGBM) proteomic model for lupus classification before the clinical diagnosis date (incident cases with respect to the proteomic profiling date) in the UK Biobank. We defined these lupus cases as (1) no self‐report of lupus or any other immunomodulatory medication use at the nurse interview during cohort enrollment (Supplementary Material 3) and (2) not having any diagnosis code of lupus in the hospital or primary care records for at least six months after the proteomic profiling. Given the challenges in determining the exact date of onset with hospital admission records, we limited the analysis to individuals who had primary care records available (~45% of the whole cohort) and had at least two lupus diagnosis codes in their linked medical records (Supplementary Material 3). We trained our models on the prevalent lupus cases using the hyperparameters identified in the main analysis and tested their performance on classifying the incident lupus cases after cohort entry. For this analysis, we randomly split the control data set (with available primary care data) into two groups, assigning one to the training set with prevalent cases and assigning the other to the independent test set, which included the incident lupus cases. The performance of the LightGBM model was then evaluated and compared to the PRS using the same strategy as outlined above.

### Ethical approval information

This research was conducted using the UK Biobank Resource under application 145447. UK Biobank has received ethical approval from the North West Multi‐Centre Research Ethics Committee (REC reference: 16/NW/0274). All participants provided informed consent at the time of enrollment.

### Data statement

This study used data from the UK Biobank Resource, available to authorized users. The mass spectrometry data are available through ProteomeXchange (PXD031979).

## RESULTS

### Demographics of lupus cases and autoimmune disease controls

After quality controls, 44,173 individuals with available proteomic data were included in the analysis. In this cohort, we identified the following numbers of prevalent cases with autoimmune diseases: 383 lupus, 670 rheumatoid arthritis, 327 multiple sclerosis, 558 inflammatory bowel disease, 135 ankylosing spondylitis, and 135 other inflammatory spondyloarthropathy. The prevalent lupus cohort had a mean age of 43.6 ± SD 11.7 years at disease onset and 56.5 ± SD 8.1 years at sample collection, with 86.2% being female and 83.3% self‐reporting being of White populations. The detailed demographics of all disease cohorts are described in Table 1.

**Table 1:** Demographics of all patients with prevalent systemic rheumatic autoimmune diseases in the UK Biobank included in this study*.

	SLE (*n* = 383)	RA (*n* = 670)	IBD (*n* = 558)	MS (*n* = 327)	AS (*n* = 135)	OS (*n* = 135)	HC (*n* = 41,998)
Age (mean ± SD)	56.5 ± 8.1	59.3 ± 7.6	58.3 ± 8.1	55.8 ± 7.7	57.9 ± 7.8	59.7 ± 7.4	57.3 ± 8.2
Female sex (*n*, %)	330 (86.2)	485 (72.4)	277 (49.6)	227 (69.4)	44 (32.6)	85 (63)	22,447 (53.4)
White (*n*, %)	319 (83.3)	618 (92.2)	544 (97.5)	320 (97.9)	131 (97)	126 (93.3)	39,070 (93)
Asian (*n*, %)	17 (4.4)	20 (3)	8 (0.7)	0 (0)	1 (0.7)	4 (3)	792 (1.9)
Black (*n*, %)	30 (7.8)	16 (2.4)	4 (1.4)	1 (0.3)	1 (0.7)	3 (2.2)	1005 (2.4)
Other populations (*n*, %)	17 (4.4)	16 (2.4)	2 (0.4)	6 (1.8)	2 (1.5)	2 (1.5)	1131 (2.7)

* 136 patients have more than one of the listed autoimmune diseases. Age is at sample collection. “Other populations” include Chinese, other ethnic group, do not know, and prefer not to answer. Age was compared across groups using analysis of variance, while sex and population (White vs non‐White) were compared using chi‐square tests. All comparisons were statistically significant (*P* value < 1 × 10^−5^). AS, ankylosing spondylitis; HC, healthy controls (individuals without any of the other autoimmune diseases listed in the table); IBD, inflammatory bowel disease; MS, multiple sclerosis; OS, other inflammatory spondyloarthropathies; RA, rheumatoid arthritis; SLE, systemic lupus erythematosus. UK Biobank survey had a separate category for self‐reported Chinese ethnic background.

### Hierarchical clustering of lupus and other autoimmune diseases based on serum proteomics

Differential proteomic expression analysis showed that there is a distinct serum proteomic signature for each autoimmune disease compared to controls (Supplementary Figure 1). Notably, lupus had the highest number of total proteins that were either overexpressed or underexpressed compared to controls among the autoimmune diseases included in the study (Supplementary Table 2). Hierarchical clustering analysis grouped lupus with rheumatoid arthritis as a distinct cluster separate from the other autoimmune diseases (Figure 1).

**Figure 1:**
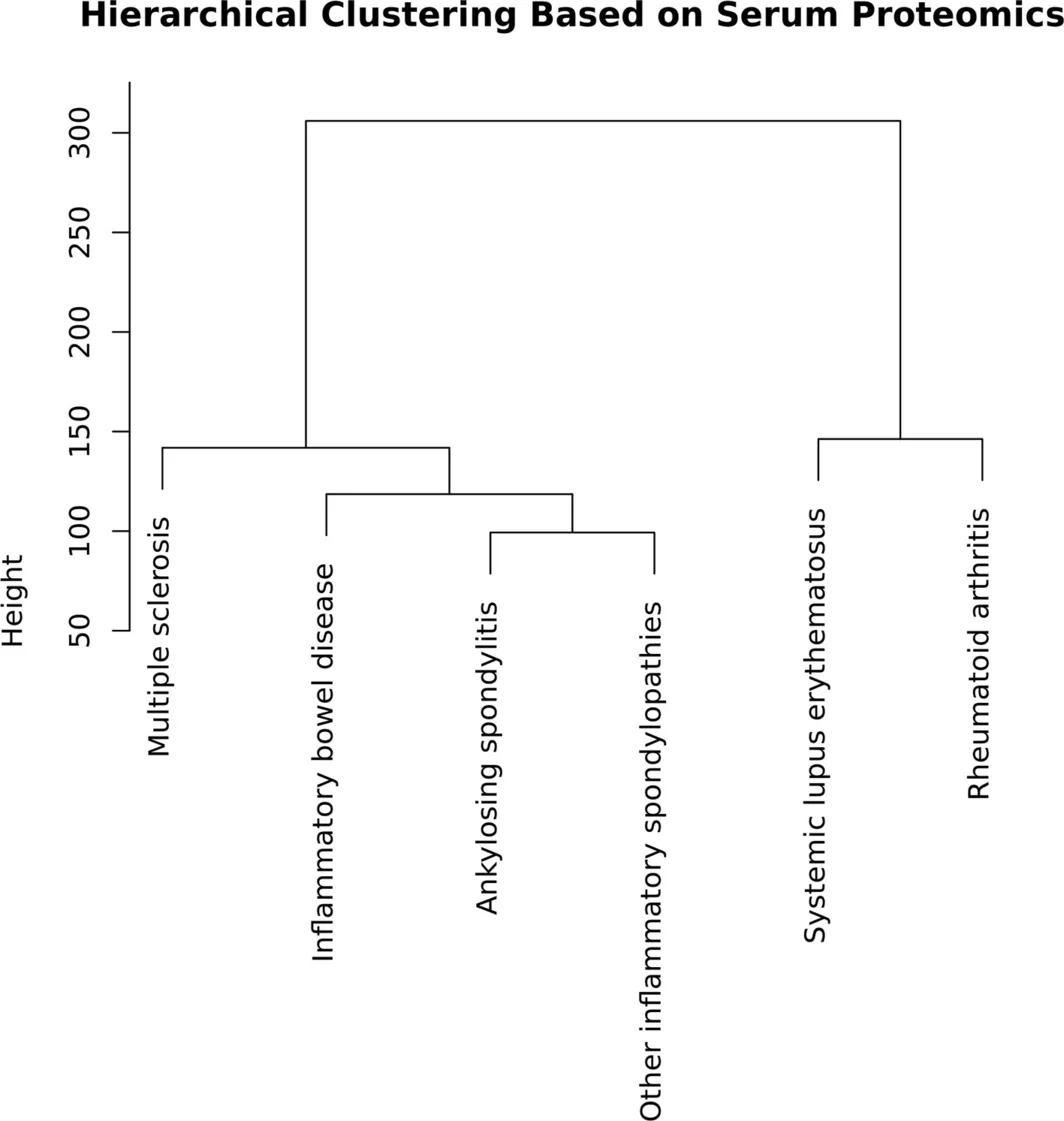
Clustering of systemic lupus erythematosus compared to other autoimmune diseases based on serum proteomic profiles. Hierarchical clustering was performed using moderated t‐statistics profiles, which quantify both the strength and direction of the association for each protein relative to controls.

### Lupus proteomic signature demonstrates generalizability across independent cohorts

Of 1,145 proteins measured in both HPA and the UK Biobank, 617 were significantly associated with lupus in HPA, and 406 were replicated in the UK Biobank (65.8% replication rate). Effect sizes (logFC) for replication candidates were also moderately correlated between the data sets (r = 0.53, *P* value = 1.9 × 10^−46^; Figure 2).

**Figure 2:**
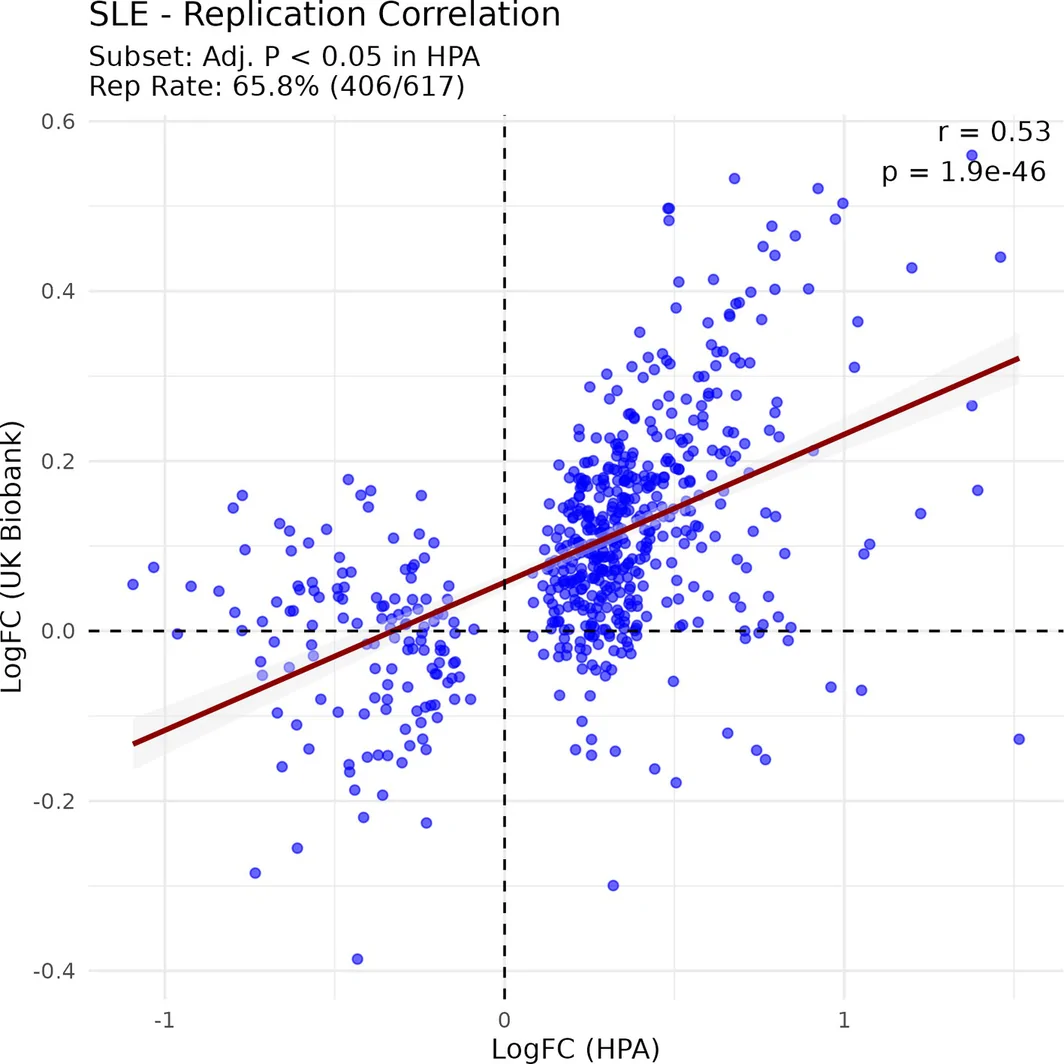
Replication rate and effect size correlation in the HPA lupus cohort compared to the UK Biobank. r and *P* value per Pearson correlation. Adj, adjusted; HPA, Human Protein Atlas; LogFC, log fold change; SLE, systemic lupus erythematosus.

### Machine learning improves proteomic classification models for lupus

Lupus had the highest performance compared to the other autoimmune diseases across all metrics using logistic regression, which represents the linear approach (Supplementary Table 3). The machine learning model (LightGBM) demonstrated further improvements in the prediction performance for lupus compared to logistic regression (ROC‐AUC mean 0.91 ± SD 0.03 vs 0.87 ± SD 0.01 and PR‐AUC mean 0.42 ± SD 0.05 vs 0.36 ± SD 0.05; Figure 3 and Supplementary Figure 2). The difference in model performances between the two models was also statistically significant (ROC‐AUC difference 0.04, 95% confidence interval [CI] 0.02–0.06, *P* value 8.2 × 10^−6^). Further, the serum proteomic model showed significantly improved performance on the immunomodulatory medication use subgroup (n = 217) compared to individuals with no self‐reported medication use (n = 166): mean ROC‐AUC of 0.98 ± SD 0.01 vs mean ROC‐AUC of 0.82 ± SD 0.06 (Supplementary Figure 3). The LightGBM model achieved a mean sensitivity of 68% ± SD 4% in the whole cohort and a mean of 87% ± SD 5% in the immunomodulatory medication use group at the 95% specificity threshold.

**Figure 3:**
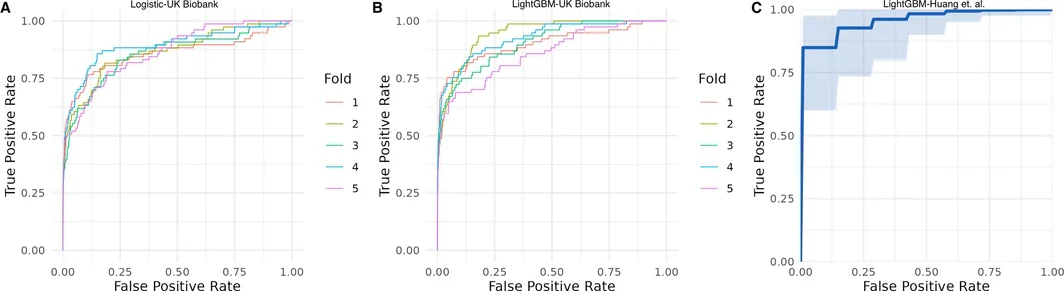
Receiver operating characteristic curves across 5‐fold cross‐validation for prevalent lupus classification for (A) logistic regression in the UK Biobank, (B) LightGBM in the UK Biobank, and (C) LightGBM in an independent lupus cohort from China. In the UK Biobank, model performance was assessed using 5‐fold stratified cross‐validation, in which the data set was partitioned into 5 subsets. In each iteration, the model was trained on 4 folds and tested on the remaining fold, with stratification by disease prevalence, age, sex, and population to ensure representative sampling. Mean values are given in the following form and calculated across 5‐fold cross‐validation: mean ± SD. For independent testing in data derived from Huang et al,^16^ the shaded blue area represents the 95% confidence interval of the receiver operating characteristic curve, estimated from 100 random splits with model retraining, using calibration and evaluation controls as described in Supplementary Material 4.

In our sensitivity analyses, the LightGBM model showed good generalization across different batches in the leave‐one‐batch‐out cross‐validation (mean ROC‐AUC and PR‐AUC across six batches were 0.92 ± SD 0.03 and 0.42 ± SD 0.05, respectively; Supplementary Table 4). Further, no significant difference was observed for model performance with the nested cross‐validation (mean ROC‐AUC 0.91 ± SD 0.02 and mean PR‐AUC 0.41 ± SD 0.05) or with missing data imputation (Supplementary Table 5). The model performance for individuals with or without primary care record–based lupus diagnoses was comparable (mean ROC‐AUC 0.90 ± SD 0.03).

In replication analysis, the LightGBM model trained on the UK Biobank cohort by using overlapping proteins (n = 380) showed excellent performance for discriminating the lupus cases from controls in the independent cross‐platform proteomic data set from Huang et al (mean ROC‐AUC 0.96 ± SD 0.03 across 100 random independent splits).

### Genetic risk stratification enhances proteome‐based disease classification

The LightGBM model had superior classification performance for lupus for individuals in the high genetic risk group compared to individuals in the low genetic risk group (mean ROC‐AUC 0.92 ± SD 0.03 vs 0.83 ± SD 0.08 and mean PR‐AUC 0.46 ± SD 0.10 vs 0.17 ± SD 0.07; Supplementary Table 6). The differences between the high and medium genetic risk groups were less profound (Supplementary Table 6). Of the 176 variants included in the lupus PRS, we identified 9 variants with significant pQTL effects in the UK Biobank, indicating that higher lupus genetic risk could exert an effect on the serum proteomic profiles (Supplementary Table 7).

### Model reverse engineering uncovers candidate biomarkers with significant effects on lupus detection

The feature rankings by the SHAP analysis showed good concordance with split and gain importance metrics from the LightGBM model (with split importance: 80% overlap on the top 20 features, Spearman correlation of 0.96; with gain importance: 50% overlap on the top 20 features, Spearman correlation of 0.81). The most influential protein was ranked as SCARB2 by both split importance and SHAP, whereas gain importance ranked Galectin‐9 as the top feature (Supplementary Figure 4). Notably, 85% of the proteins had nonzero mean absolute SHAP values, indicating a contribution to the model prediction, whereas for the logistic regression, 33% of the proteins had nonzero average coefficients across all folds (Supplementary Tables 8 and 9).

Empirical validation of 32 proteins ranked within the top 20 by at least one of these metrics through the leave‐one‐feature‐out approach showed that SCARB2 had the strongest effect on model performance with a mean PR‐AUC drop of 0.024 ± SD 0.003 followed by SOD2 (mean 0.021 ± SD 0.007), CD302 (mean 0.011 ± SD 0.007), and GGT5 (mean 0.011 ± SD 0.007) proteins (Figure 4). These four proteins only had a modest increase in their mean expression levels in lupus compared to controls, and except for CD302, none were ranked in the top 20 strongest associations per the *P* value rankings in the differential expression analysis (Supplementary Table 2 and Supplementary Figure 5).

**Figure 4:**
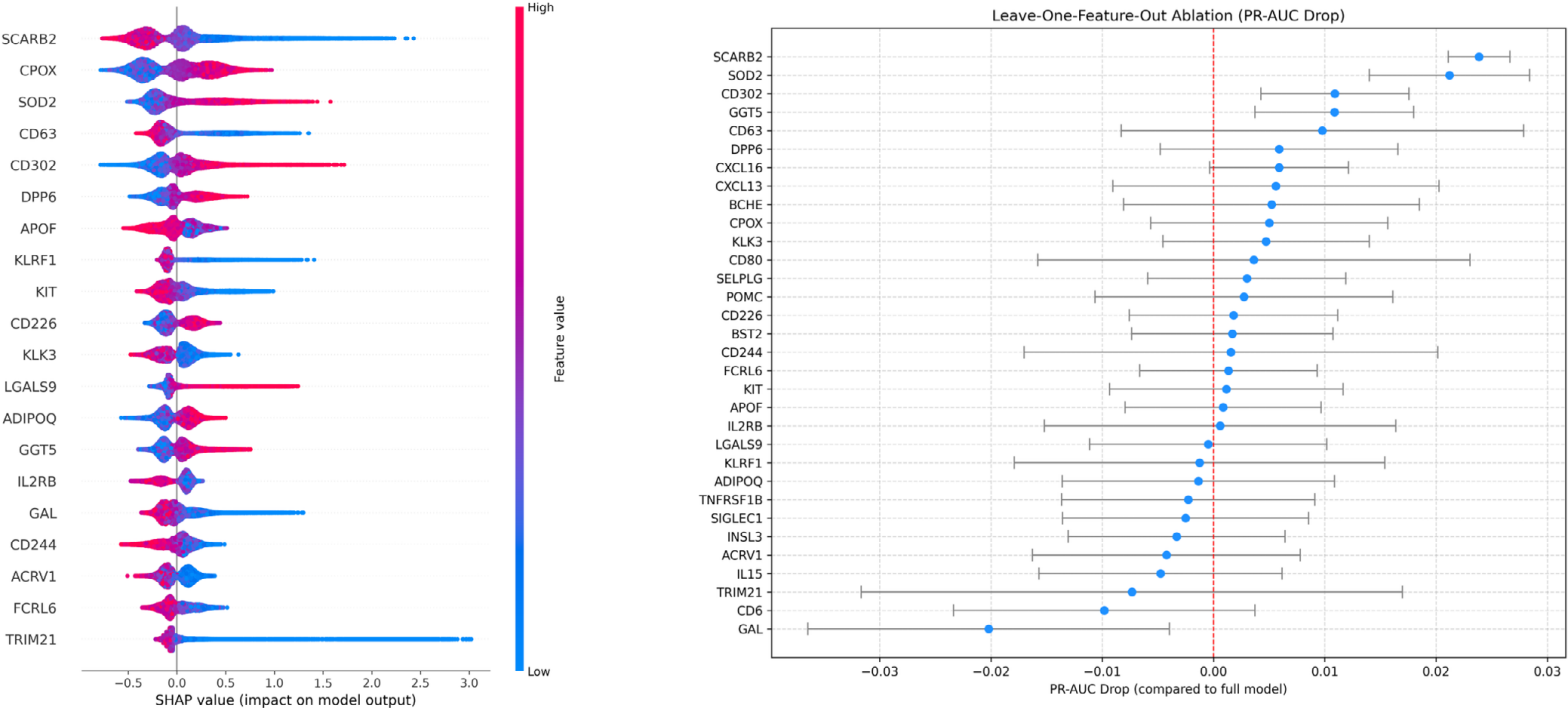
Top proteins influencing LightGBM model performance ranked by their importance as assessed by drop in the PR‐AUC, using SHAP and leave‐one‐feature‐out analysis. (Left) SHAP beeswarm plot: The x‐axis depicts SHAP importance values (impact on model output) with positive values influencing the model prediction toward the positive class (lupus). The y‐axis depicts the features sorted by overall SHAP importance. Each sample is depicted as a dot. The color of each dot represents the original feature value with lower values depicted as blue and higher values depicted as red. (Right) Leave‐one‐feature‐out ablation results: Blue dots indicate the mean PR‐AUC drop across 5‐fold cross‐validation and the bars indicate its SD. The red dashed line represents no change in PR‐AUC between the full model and ablated model. Negative PR‐AUC drop values indicate a gain in model performance by dropping the relevant feature. ACRV1, acrosomal protein; ADIPOQ, adiponectin; APOF, apolipoprotein F; BCHE, cholinesterase; BST2, bone marrow stromal antigen 2; CD6, T cell differentiation antigen CD6; CD63, AD1 antigen; CD80, T‐lymphocyte activation antigen CD80; CD226, CD226 antigen; CD244, natural killer cell receptor 2B4; CD302, C‐type lectin domain family 13 member A; CPOX, oxygen dependent coproporphyrinogen III oxidase; CXCL13, C‐X‐C motif chemokine 13; CXCL16, C‐X‐C motif chemokine 16; DPP6, dipeptidyl aminopeptidase‐like protein 6; FCRL6, Fc receptor like protein 6; GAL, galanin peptides; GGT5, glutathione hydrolase 5 proenzyme; IL2RB, interleukin 2 receptor subunit beta; IL15, interleukin 15; INSL3, insulin like 3; KIT, mast/stem cell growth factor receptor; KLK3, prostate specific antigen; KLRF1, killer cell lectin like receptor subfamily F member 1; LGALS9, Galectin 9; POMC, pro‐opiomelanocortin; PR‐AUC, area under the precision‐recall curve; SCARB2, lysosome membrane protein 2; SELPLG, P‐selectin glycoprotein ligand 1; SHAP, Shapley additive explanations; SIGLEC1, sialoadhesin; SOD2, superoxide dismutase 2; TNFRSF1B, tumor necrosis factor receptor superfamily member 1B; TRIM21, E3 ubiquitin protein ligase.

### Proteomics model shows promising performance for classifying lupus before clinical diagnosis

For incident lupus subgroup analysis, 14 cases were identified. The cases had a mean age of 59.6 ± SD 8.1 years at sample collection with 78.6% being female and 92.9% self‐reporting being of White populations. Proteomic profiling preceded the clinical lupus diagnosis by an average of 3.4 ± SD 2.1 years. The LightGBM model achieved an ROC‐AUC of 0.78 (95% CI 0.62–0.94) for incident lupus cases while the PRS achieved an ROC‐AUC of 0.67 (95% CI 0.62–0.71) in all lupus cases in the UK Biobank (Figure 5).

**Figure 5:**
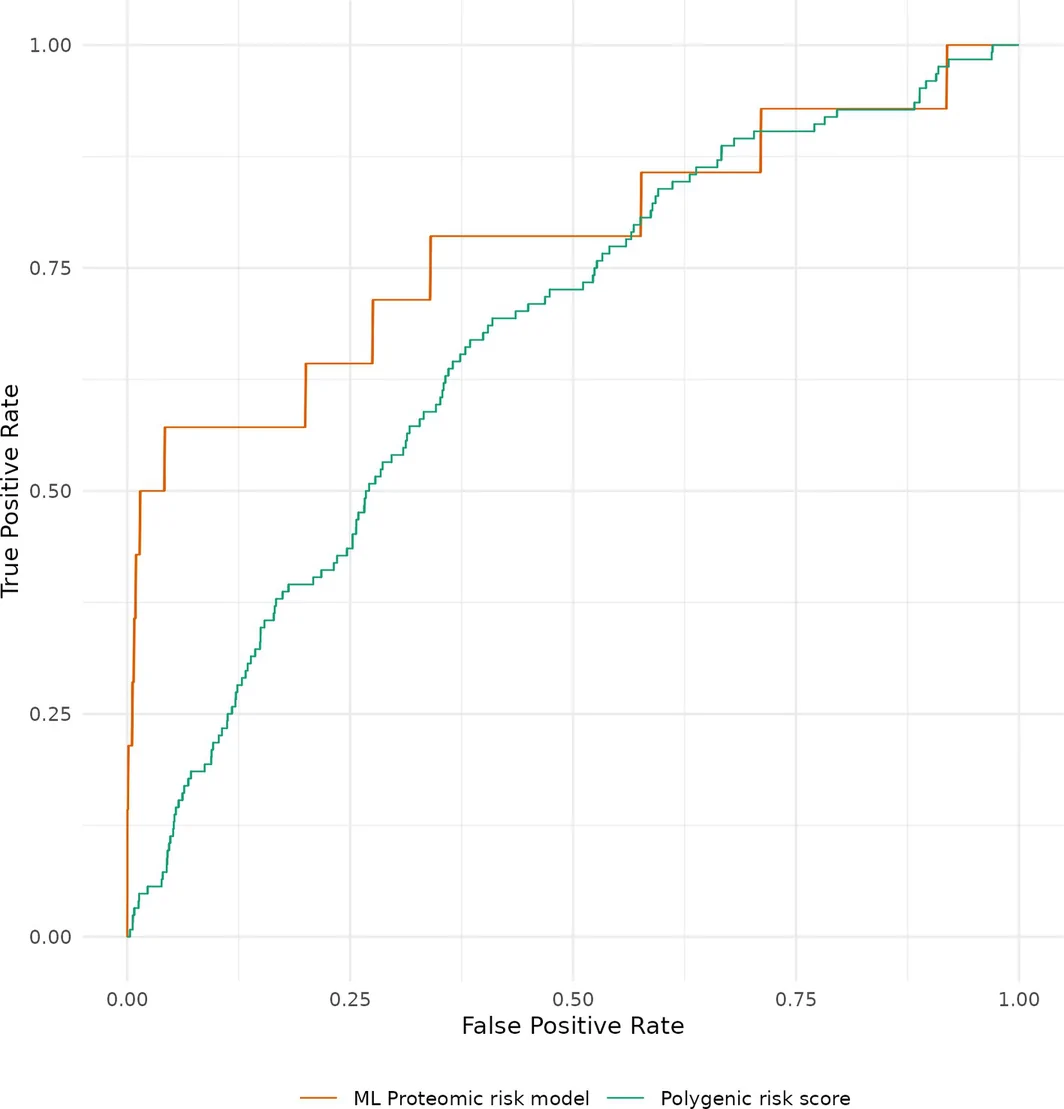
Receiver operating characteristic curves for the LightGBM model in individuals with incident lupus and polygenic risk score calculated using all lupus cases in the UK Biobank. ML, machine learning.

## DISCUSSION

In this study, we demonstrate that machine learning–based proteomic classification models achieve excellent performance for identifying pre‐existing lupus across independent cohorts and proteomic profiling platforms. Further, we show that the proteomic models perform well for identifying lupus before clinical diagnosis. We also show that for individuals at high genetic risk, serum proteomic models have better prediction accuracy compared to individuals who are at low genetic risk. Further, we identify influential candidate biomarkers for lupus through reverse engineering the machine learning model beyond those highlighted by the case‐control analysis. Our results underscore the significant potential for proteome‐based disease classification and risk prediction in lupus.

Existing literature on proteomic risk assessment in lupus has focused on selecting a limited number of protein biomarkers using various feature selection methods and testing their performance for predicting future lupus risk, with generally low to modest accuracy.^10^, ^11^ A prior analysis that studied the predictive performance of a five‐protein panel for a 10‐year incidence rate of 218 diseases, including lupus, reported a low accuracy for predicting lupus incidence (C‐index: 0.64, 95% CI 0.55–0.73).^11^ Another analysis on a small subset of UK Biobank data, which focused only on women with incident lupus and using a 22‐protein panel, reported an ROC‐AUC of 0.82 with very wide CIs (95% CI 0.54–0.99).^10^ In contrast, our results demonstrate that comprehensive serum proteomic models leveraging machine learning could have significantly higher performance (mean ROC‐AUC 0.91 ± SD 0.03) for identifying established lupus, achieving an overall sensitivity of ~70% at 95% specificity. For individuals with self‐reported immunomodulatory medication use, the performance was even greater (mean ROC‐AUC 0.98 ± SD 0.01) corresponding to ~90% sensitivity at 95% specificity. This performance was further replicated in an independent lupus cohort from China that underwent proteomic profiling with mass spectrometry.

This strong replication, as well as the partial reproducibility of the lupus proteomic risk signature between independent datasets, suggests that variability in the proteome, although dynamic, could be persistent and reproducible in lupus. Hence, developing proteome‐based lupus diagnostic tests could be a feasible strategy. Integrating this into a clinical workflow will require validating the model performance at the time of diagnosis but before treatment, as well as considering the cost and widespread availability of proteomic profiling methods. However, the fact that proteomic models could perform well across two different platforms suggests that cheaper and targeted assays could be developed for a more limited number of proteins. Further research will also be needed to establish whether proteomic classification models used in conjunction with existing autoantibody testing frameworks could further enhance model performance. Our results also establish a proof‐of‐concept that there is potential for improved proteomic classification models through machine learning approaches. With advances in machine learning methods^35^ and growing data sets across the world through large biobanking efforts,^17^, ^36^, ^37^, ^38^ further gains in model performance and generalizability could be possible, which will be an important future research direction.

The lupus signature identified from established lupus exhibited good accuracy for classifying lupus cases before clinical diagnosis with numerically higher performance compared to PRS. This suggests that at least a significant portion of the lupus‐associated proteome change occurs before diagnosis, which supports the observation of gradual development of autoantibodies before disease onset in lupus.^39^ These data indicate a potential future role for proteomic prediction models in lupus prevention and early diagnosis. However, further research will be needed to validate this in other cohorts and develop models quantifying time‐dependent lupus risk in the population.

We observed that several lupus genetic risk loci influence serum protein profiles through pQTL effects,^33^ which could contribute to the superior prediction performance of proteomic models in the higher genetic risk group. Further, proteomic profiling might act as proxy for environmental components of disease risk and capture the substantial environmental effects on lupus development, which are estimated to be around 56% in prior research.^40^ Hence, when integrated with genetic risk, serum proteomic models could capture potentially additive or synergistic relationships between genetic susceptibility and environmental exposure as demonstrated by the superiority of integrative genetic–environmental models compared to genetic only models.^6^, ^8^

We observed that lupus is characterized by a more pronounced proteomic dysregulation relative to several other autoimmune diseases, which is consistent with the superior performance of proteomic models in lupus. Hierarchical clustering further demonstrated that lupus and rheumatoid arthritis formed a distinct subgroup, whereas inflammatory bowel disease, multiple sclerosis, and spondyloarthropathies clustered separately, suggesting that “seropositive” diseases such as lupus and rheumatoid arthritis could share proteomic alterations that are distinct from those underlying “seronegative” conditions. These findings indicate that proteomic signatures could potentially capture both shared and disease‐specific changes across different autoimmune diseases. Further research is needed to understand whether prediction models developed across multiple autoimmune diseases could outperform disease‐specific models by leveraging the shared proteomic signatures of autoimmunity combined with the unique signature of each disease.

Reverse engineering the machine learning models identified several proteins as influential features despite only having a mild difference in their mean relative expression between cases and controls. Prior research suggests strong biologic plausibility for a potential role of these proteins in lupus. For example, the top hit SCARB2, also known as lysosome membrane protein 2, is a type III membrane protein residing in the membrane of late endosomes and lysosomes.^41^ It was shown to be required for TLR‐9‐dependent interferon production during CpG DNA stimulation in plasmacytoid dendritic cells, potentially linking it with the well‐recognized interferon signature in lupus.^42^ Further, SCARB2 was shown to have increased gene expression in peripheral blood mononuclear cells (PBMCs), glomeruli, and renal tubules of individuals with lupus nephritis (LN) as well as in the monocyte‐derived macrophages from the kidney of patients with LN, indicating a potential role in organ involvement.^43^, ^44^, ^45^ The second top hit SOD2 is a mitochondrial protein also known as manganese‐dependent superoxide dismutase.^46^ SOD2 was shown to be overexpressed in PBMCs from patients with lupus,^47^ which is potentially linked with the mitochondrial dysfunction observed in lupus T cells.^48^ Interestingly, antibodies against SOD2 were observed in patients with LN and showed a degree of correlation with disease activity.^49^ Hence, our findings suggest that machine learning could help in the discovery of candidate biomarkers with strong biologic plausibility; however, it is worth noting that a causal role for these proteins cannot be established through our analysis alone. Moreover, it is notable that dropping individual proteins only had a modest effect on the model performance, indicating that comprehensive assessment of the proteome is necessary for high‐performing proteomic models.

This study has several notable strengths. First, we developed and replicated proteomic models for lupus classification with strong performance, demonstrating the potential cross‐cohort, cross‐platform generalizability of the lupus proteomic signature. Second, we demonstrated that machine learning could improve proteomic models for lupus, which is an underexplored research area. Third, we demonstrated a proof‐of‐concept for the superior prediction potential of genetic risk stratification in lupus, suggesting the promise of integrative prediction models across multi‐omics layers. Fourth, we showed that reverse engineering machine learning models could help identify influential biomarkers not strongly highlighted in the traditional case‐control analyses, which is a novel concept in lupus research.

This study has several limitations. The UK Biobank lupus cohort included in our analysis was identified through clinical codes in the national databases of the UK; hence, misclassification bias is possible. However, when the analysis was restricted to a more specific lupus definition by including immunomodulatory medication use, we observed superior model performance, suggesting that potential misclassification likely leads to an underestimation of model performance rather than an overestimation, which was further confirmed in an independent cohort. Nevertheless, proteomic profiling before treatment will be necessary to rule out potential confounding effects of immunomodulatory therapy. Due to incomplete primary care record availability in the UK Biobank, it is possible that some incident cases are not captured. Further, it is possible that not all medical records of participants (such as private care) were linked, potentially introducing uncertainty to lupus date of onset. However, the dates of lupus diagnoses were assessed through three independent sources (primary care records, hospital admission, and self‐report during nurse interview), suggesting that only individuals with existing lupus diagnoses who deny their diagnosis or medication use at enrollment and do not have any recorded diagnoses in primary care or hospital admission records could have been misclassified as incident. The limited clinical phenotyping available precluded the evaluation of our model in different clinical lupus subtypes such as different organ involvement or autoantibody profiles in which model performances might differ. The feature importance analyses need independent testing and functional validation to establish a causal role in disease biology. Moreover, given that SHAP‐based interpretations can be influenced by multicollinearity, other proteins that are highly correlated with the identified hits may also play important roles. Although the proteomic profiling was comprehensive in this data set, not all serum proteins were measured, and only a subset of proteins was available for all cohorts. Our approach of using median‐shift alignment and missingness estimation from a small calibration set may be insufficient for nonlinear or batch‐specific platform effects within mass spectrometry data. The lupus cases in our cohort were predominantly of self‐reported White populations, reflecting the overall demographics of the UK Biobank. Although independent replication in a cohort of Chinese origin was performed, further research will be needed to expand these findings in individuals with lupus from other populations.

In conclusion, machine learning–based proteomic models show excellent performance in identifying pre‐existing lupus and generalize well to predicting individuals with future lupus diagnoses. Notably, machine learning approaches can identify candidate biomarkers with strong biologic plausibility that are not captured by traditional case‐control analyses.

## AUTHOR CONTRIBUTIONS

All authors contributed to at least one of the following manuscript preparation roles: conceptualization AND/OR methodology, software, investigation, formal analysis, data curation, visualization, and validation AND drafting or reviewing/editing the final draft. As corresponding author, Dr Sawalha confirms that all authors have provided the final approval of the version to be published and takes responsibility for the affirmations regarding article submission (eg, not under consideration by another journal), the integrity of the data presented, and the statements regarding compliance with institutional review board/Declaration of Helsinki requirements.

## Supporting information


**Disclosure form**.

**Supplementary Figure 1:** Volcano plots depicting the log fold changes and p values for case control analysis of each autoimmune disease included in the study.
**Supplementary Figure 2:** Precision‐recall curves of LightGBM and logistic regression models for lupus prediction across 5‐fold cross‐validation.
**Supplementary Figure 3:** Receiver operating characteristics (ROC) curves for lupus subgroups based on immunomodulatory medication use.
**Supplementary Figure 4:** Top 20 features per the gain and split importance metrics of the LightGBM model.
**Supplementary Figure 5:** Violin plots of the raw NPX value distribution of proteins found to have high influence on prediction performance during model interpretation analyses
**Supplementary Table 3:** Mean performance metrics of hyperparameter‐optimized logistic regression model for systemic lupus erythematosus compared to other autoimmune diseases across 5‐fold cross validation
**Supplementary Table 4:** Leave‐one‐batch‐out cross‐validation results for the LightGBM model
**Supplementary Table 5:** LightGBM model performance with two different missing data approaches.
**Supplementary Table 6:** Mean performance metrics of LightGBM models for prevalent systemic lupus erythematosus stratified based on genetic risk.

**Supplementary Table 1:** List of genetic risk variants used in the construction of polygenic risk score

**Supplementary Table 2:** Differential proteomic expression analysis results for autoimmune diseases included in the study

**Supplementary Table 7:** List of lupus PRS genetic variants with pQTL effects in the UK Biobank across all populations

**Supplementary Table 8:** List of proteins with non‐zero mean coefficients across 5 folds in logistic regression

**Supplementary Table 9:** Mean absolute SHAP values for each protein across all samples and folds
