# Supplementary material for: Identifying Systemic Lupus Erythematosus From Serum Proteomic Profiles Using Machine Learning and Genetic Risk Stratification
Source: Arthritis Rheumatol. 2026 May 4;78(11):2280–91. doi: 10.1002/art.70156 (PMC13712558; doi:10.1002/art.70156)
Supplement: Supplementary file 2 [file ART-78-2280-s005.docx]

**Supplementary Data**

**Supplementary Material 1.** For case selection, we utilized the first occurrences data release of the UK Biobank (Category 1712) which mapped the disease codes across the primary care, hospital inpatient, death register and self-reported diagnoses to 3-character ICD-10 codes. The details between the diagnostic mapping between the different diagnostic codes and disease labels were provided in the following resource: https://biobank.ndph.ox.ac.uk/showcase/refer.cgi?id=593.

For proteomics data, we utilized the preprocessed Olink Proteomics data which underwent comprehensive quality controls by the UK Biobank Pharma Proteomics Project (Category 1839). The details regarding these quality controls steps are provided in the <https://biobank.ndph.ox.ac.uk/showcase/refer.cgi?id=4658> (Resource 4658). The details regarding the Normalized Protein eXpression (NPX) calculation for the Olink platform are provided in the following UK Biobank document (Resource 2057): <https://biobank.ndph.ox.ac.uk/ukb/refer.cgi?id=2057>.

**Supplementary Material 2.** For logistic regression hyperparameter optimization process, we performed a grid search over both L1 and L2 parameters separately for the following C values: 0.0001, 0.001, 0.01, 0.1, 1 (no regularization). For LightGBM hyperparameter optimization, we initially performed a random search over the following hyperparameters: learning_rate, which controls step-size shrinkage; num_leaves, the maximum number of leaves per tree; min_child_samples, the minimum data points in a leaf; and min_split_gain, the minimum loss reduction for a partition, along with n_estimators, the number of boosting trees, and reg_lambda, the L2 regularization penalty. This was followed by a Bayesian search to refine the most important parameters. Weights & Biases (wandb) package was used for hyperparameter optimization. Random grid search was performed for 50 iterations, while the subsequent Bayesian search was performed for 10 additional iterations.

The best hyperparameters identified for logistic regression were L1 penalty with C value of 0.1 for lupus, ulcerative colitis, multiple sclerosis; L1 penalty with C value of 0.01 for rheumatoid arthritis; L1 penalty with C value of 0.001 for ankylosing spondylitis and other inflammatory spondyloarthropathies; L2 penalty with value of 0.01 for Crohn`s disease.

The best hyperparameters for LightGBM for lupus were the following: number of estimators: 300, number of leaves: 14, min split gain: 0.2, reg lambda: 5, min child sample 150, with the remaining parameters used as their default values. “Column sample by tree” and “subsample” parameters (which randomly excludes columns or samples during training) were not tuned to ensure stability across leave-one-feature-out and sample subgroup analyses. In the sensitivity analysis with the nested cross-validation, we performed 50 iterations with random grid search using the same hyperparameter specifications.

**Supplementary Material 3.** List of medications used to define immunomodulatory medication use in the UK Biobank.

1. Hydroxychloroquine (also reported as Plaquenil 200 mg tablet)
2. Methotrexate
3. Prednisone (also reported as prednisolone, methylprednisolone and prednisolone product)
4. Mycophenolate
5. Azathioprine (also reported as Imuran)
6. Leflunomide (also reported as Arava 10 mg tablet or Arava 20 mg tablet)
7. Cyclophosphamide

These medication data were obtained through a verbal interview by a trained nurse at cohort entry. The detailed description of these data can be found at the following resource: <https://biobank.ndph.ox.ac.uk/ukb/label.cgi?id=100075> (Category 100075). The positive and negative predictive value of a self-reported medication compared to the primary care prescription records (available in 45% of the cohort) are shown in the following table:

| **Drug** | **Positive Predictive Value** | **Negative Predictive Value** |
| --- | --- | --- |
| Methotrexate | 77.87% | 99.78% |
| Hydroxychloroquine | 84.51% | 99.71% |
| Prednisone | 86.36% | 90.16% |
| Leflunomide | 77.78% | 99.92% |
| Azathioprine | 78.46% | 99.62% |
| Mycophenolate | 77.78% | 99.92% |

For identifying the prescription records for the same list of medications, we searched for them individually (including aliases/brand names) in the Drug Name column for the prescription records provided by TPP (company) for England. As the mapping for drug name was not available for other countries in the UK, they were excluded from the analysis. The details of the primary care record data capture strategy and its limitations are described in detail at the following resource: <https://biobank.ndph.ox.ac.uk/showcase/refer.cgi?id=591> (Resource 591).

For identifying incident lupus cases in the primary care records from the UK Biobank, we utilized the primary care read codes (Read v2 and CTV3 (Read v3) codes). We utilized the mapping with ICD10 codes and primary care read codes provided by the UK Biobank to identify lupus read codes: <https://biobank.ndph.ox.ac.uk/showcase/ukb/auxdata/primarycare_codings.zip>.

**Supplementary Material 4.** Control-based alignment in the mass spectrometry dataset from Huang et. al.

We randomly selected 3 controls from the Huang et. al. dataset as calibration controls with the rest reserved as evaluation controls. For each protein, we computed the median expression value in these calibration controls, and also in the Olink data from all participants in the UK Biobank. We then transformed all remaining samples from the Huang et. al. dataset (evaluation controls and cases) by median shifting to the Olink distribution. To align the platform dependent missingness between the two cohorts, we estimated the missingness proportion in the Huang et. al. calibration controls for each protein and simulated their left-censoring in the UK Biobank by setting the lowest corresponding proportion of UK Biobank values to missing. The missing values were not imputed to minimize data transformations and instead were processed by the LightGBM during model training. Further, to estimate the variance of the control-based alignment, we randomly selected the calibration set (n=3) from all Huang et. al. controls followed by model retraining and evaluation for 100 repeated random splits. ROC-AUC performance metric was then reported as mean ± SD. The calibration set was excluded from the model evaluation in each iteration. As the total control sample size in Huang et. al. was small (n=10), we compared the mean log-transformed Label-Free Quantification (LFQ) intensities of the controls against plasma protein concentrations reported in the large independent HPA cohort (**figure above**). We observed a strong correlation between log means of the Huang et. al. control samples and the HPA cohort samples (Pearson correlation 0.84, R^2^ 0.71). This supports that the proteomic abundances in this limited dataset are consistent with the general population supporting the validity of the control-based calibration approach that we employed.

We further validated our approach by stratifying protein missingness in our Huang et. al. data against the HPA cohort. We observed that 81% of missing values occurred in proteins within the lowest quartile of population abundance, whereas high-abundance proteins exhibited less than 10% missingness (**figure above**). This indicates that missing data in the Huang et. al. data is predominantly driven by low protein abundance, consistent with a left-censoring (limit-of-detection) mechanism. This confirms the need to align the platform dependent missingness, given the significantly lower limit of detection in Olink compared to mass spectrometry.

Controls, Huang et. al. control samples; Reference, HPA samples; Lupus Cases, Huang et. al. lupus samples.

**Supplementary Figure 1.** Volcano plots depicting the log fold changes and p values for case control analysis of each autoimmune disease included in the study.

Significance defined as having an adjusted p-values less than 0.05 and a log fold difference NPX value of ± 0.14 corresponding to 10% difference as NPX values are on log2 scale. Significance hits are bolded in orange. Top 5 strongest associations were annotated for each disease.

**Supplementary Figure 2.** Precision-recall curves of LightGBM and logistic regression models for lupus prediction across 5-fold cross-validation.

PR-AUC, Area under the curve of precision-recall curve. Mean ± SD across 5-fold cross-validation.

**Supplementary Figure 3.** Receiver operating characteristics (ROC) curves for lupus subgroups based on immunomodulatory medication use.

ROC curves for each fold of 5-fold cross-validation depicted separately for individuals who have immunomodulatory medication prescription in their records (ROC-AUC of 0.98 ± 0.01, blue) and those who do not (ROC-AUC of 0.82 ± 0.06, red).

**Supplementary Figure 4.** Top 20 features per the gain and split importance metrics of the LightGBM model.

**Supplementary Figure 5**. Violin plots of the raw NPX value distribution of proteins found to have high influence on prediction performance during model interpretation analyses

Red bar indicates mean and standard deviation.

**Supplementary Table 3.** Mean performance metrics of hyperparameter-optimized logistic regression model for systemic lupus erythematosus compared to other autoimmune diseases across 5-fold cross validation

|  | **ROC-AUC**  (mean±SD, min-max) | **PR-AUC**  (mean±SD, max-min) | **Max F1 Score**  (mean±SD, max-min) |
| --- | --- | --- | --- |
| Systemic lupus erythematosus | 0.87 ± 0.01 (0.85-0.89) | 0.36 ± 0.05 (0.30-0.42) | 0.43 ± 0.03 (0.39-0.46) |
| Rheumatoid arthritis | 0.81 ± 0.02 (0.78-0.83) | 0.23 ± 0.04 (0.17-0.29) | 0.31 ± 0.06 (0.24-0.39) |
| Inflammatory bowel disease | 0.79 ± 0.02 (0.77-0.82) | 0.16 ± 0.02 (0.14-0.19) | 0.25 ± 0.01 (0.24-0.26) |
| Multiple sclerosis | 0.76 ± 0.03 (0.73-0.80) | 0.07 ± 0.02 (0.05-0.10) | 0.17 ± 0.03 (0.13-0.21) |
| Ankylosing spondylitis | 0.74 ± 0.04 (0.70-0.79) | 0.02 ± 0.02 (0.01-0.05) | 0.05 ± 0.03 (0.03-0.09) |
| Other spondyloarthropathies | 0.59 ± 0.06 (0.51-0.65) | 0.01 ± 0.00 (0.00-0.01) | 0.04 ± 0.02 (0.01-0.06) |

AUC, Area under the curve. PR, Precision-Recall. ROC, Receiver operating characteristics. Max, Maximum. Min, Minimum. Mean ± standard deviation (SD) and max-min per 5-fold cross-validation.

**Supplementary Table 4**. Leave-one-batch-out cross-validation results for the LightGBM model

| **Train Batch** | **Test Batch** | **ROC-AUC** | **PR-AUC** | **Max F1** |
| --- | --- | --- | --- | --- |
| 2,3,4,5,6 | 1 | 0.9614 | 0.3985 | 0.4571 |
| 1,3,4,5,6 | 2 | 0.9236 | 0.4923 | 0.5397 |
| 1,2,4,5,6 | 3 | 0.8910 | 0.4414 | 0.4867 |
| 1,2,3,5,6 | 4 | 0.8851 | 0.3392 | 0.3704 |
| 1,2,3,4,6 | 5 | 0.9116 | 0.4301 | 0.4633 |
| 1,2,3,4,5 | 6 | 0.9213 | 0.4348 | 0.4724 |

Batch 0 was removed from batch specific analyses as there were only 69 individuals processed in that batch. AUC, Area under the curve. PR, Precision-Recall. ROC, Receiver operating characteristics.

**Supplementary Table 5**. LightGBM model performance with two different missing data approaches.

|  | **ROC-AUC**  (mean±SD, min-max) | **PR-AUC**  (mean±SD, max-min) | **Max F1 Score**  (mean±SD, max-min) |
| --- | --- | --- | --- |
| Median Imputation | 0.91 ± 0.03 (0.86-0.94) | 0.42 ± 0.05 (0.38-0.49) | 0.46 ± 0.04 (0.43-0.52) |
| No imputation | 0.90 ± 0.03 (0.85-0.93) | 0.42 ± 0.05 (0.38-0.49) | 0.46 ± 0.05 (0.42-0.53) |

AUC, Area under the curve. PR, Precision-Recall. ROC, Receiver operating characteristics. Max, Maximum. Min, Minimum. Mean ± standard deviation (SD) and max-min per 5-fold cross-validation.

**Supplementary Table 6.** Mean performance metrics of LightGBM models for prevalent systemic lupus erythematosus stratified based on genetic risk.

|  | **ROC-AUC**  (mean±SD, min-max) | **PR-AUC**  (mean±SD, min-max) | **Max F1 Score**  (mean±SD, min-max) |
| --- | --- | --- | --- |
| High genetic risk | 0.92 ± 0.03 (0.90-0.96) | 0.46 ± 0.10 (0.34-0.60) | 0.50 ± 0.09 (0.43-0.60) |
| Medium genetic risk | 0.88 ± 0.03 (0.84-0.91) | 0.34 ± 0.09 (0.23-0.47) | 0.44 ± 0.07 (0.36-0.55) |
| Low genetic risk | 0.83 ± 0.08 (0.70-0.91) | 0.17 ± 0.07 (0.09-0.28) | 0.29 ± 0.07 (0.18-0.38) |

AUC, Area under the curve. PR, Normalized Precision-Recall. ROC, Receiver operating characteristics. Max, Maximum. Mean ± standard deviation (SD) and max-min per 5-fold cross-validation.
